# The role of chitin, chitinases, and chitinase-like proteins in pediatric lung diseases

**DOI:** 10.1186/s40348-015-0014-6

**Published:** 2015-02-27

**Authors:** Ines Mack, Andreas Hector, Marlene Ballbach, Julius Kohlhäufl, Katharina J Fuchs, Alexander Weber, Marcus A Mall, Dominik Hartl

**Affiliations:** Department of Pediatrics/UKBB, University of Basel, Petersplatz 1, 4003 Basel, Switzerland; Children’s Hospital, University of Tübingen, Hoppe-Seyler-Strasse 1, 72076 Tübingen, Germany; Interfaculty Institute for Cell Biology, Department of Immunology, University of Tübingen, Geschwister-Scholl-Platz, 72074 Tübingen, Germany; Department of Translational Pulmonology, Division of Pediatric Pulmonology and Allergy and Cystic Fibrosis Center, Translational Lung Research Center Heidelberg (TLRC), Member of the German Center for Lung Research (DZL), University of Heidelberg, Grabengasse 1, 69117 Heidelberg, Germany

**Keywords:** Inflammation, Lung diseases, Asthma, Cystic fibrosis, Chitin, Chitinases, Chitinase-like proteins, Chitotriosidase, AMCase, CHIT1, YKL-40, BRP-39, Fungi, M2 macrophages

## Abstract

Chitin, after cellulose, the second most abundant biopolymer on earth, is a key component of insects, fungi, and house-dust mites. Lower life forms are endowed with chitinases to defend themselves against chitin-bearing pathogens. Unexpectedly, humans were also found to express chitinases as well as chitinase-like proteins that modulate immune responses. Particularly, increased levels of the chitinase-like protein YKL-40 have been associated with severe asthma, cystic fibrosis, and other inflammatory disease conditions. Here, we summarize and discuss the potential role of chitin, chitinases, and chitinase-like proteins in pediatric lung diseases.

## Introduction

**Figure 1:**
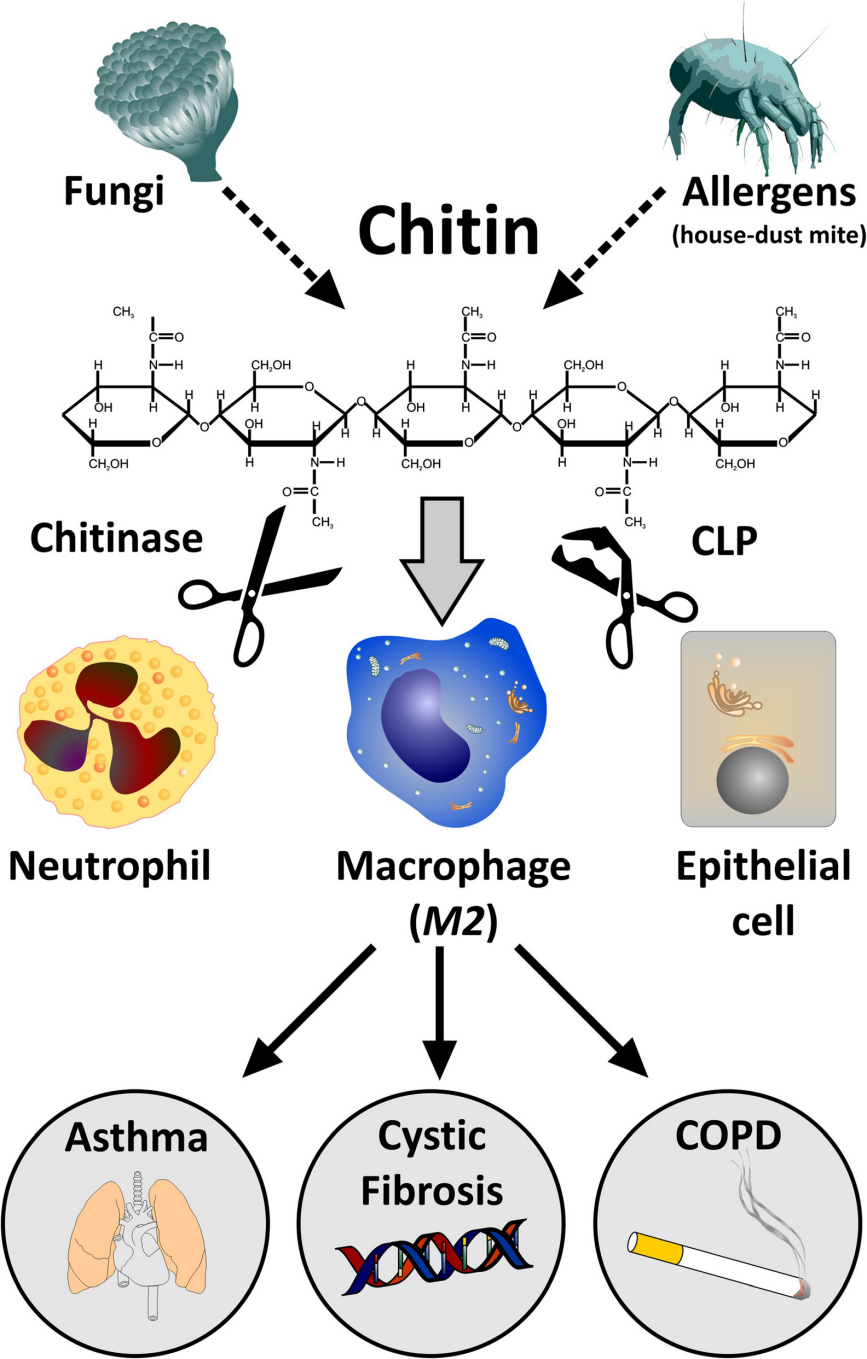
**The proposed role of chitin, chitinases and chitinase-like proteins (CLPs) in lung diseases**. Chitin is a common component of allergy-triggering environmental components, including fungal spores and house-dust mites, which trigger an innate immune response, including chitinases (cleaving chitin; scissors) and chitinase-like proteins (binding, but not cleaving chitin; damaged scissors). Chitinases and chitinase-like proteins are mainly secreted by neutrophils, alternatively activated macrophages (M2 macrophages) and epithelial cells. The interplay of M2 macrophages, neutrophils, and epithelial cells drives inflammation and remodeling in chronic lung diseases, particularly asthma, cystic fibrosis, and COPD.

The role of chitin and chitinases has been firmly established in the field of plant and microbial immunity by demonstrating that host-derived chitinases cleave chitin to protect against invading chitin-bearing pathogens, such as fungi. Although mammals lack endogenous chitin or chitin synthases, chitinases and chitinase-like proteins are endogenously expressed in their lung and other organs. Particularly, chitinase-like proteins have been described as dysregulated in a variety of diseases characterized by chronic inflammation and tissue remodeling, yet their potential role for humans has just recently begun to evolve [1,2]. Chitin is a major component of a variety of allergy-triggering environmental components, including house-dust mites or fungal spores, and fungal asthma is increasingly appreciated as an under-diagnosed disease entity [3]. Thus, an understanding of the complex immunological and pathophysiological implications of chitin-chitinase interactions in the human body is of high relevance for identifying new biomarkers and therapeutic targets for fungal diseases and other conditions, where chitin-coated microbial derivatives play a critical role. Here, we provide an overview of an emerging, yet complex field of research. Of particular interest are interspecies differences with resulting specific nomenclatures. Subsequent to an overall introduction of chitin, the role of chitinases and chitinase-like proteins in pediatric lung diseases are reviewed, leading up to a summary of ideas how these mechanisms could be exploited to improve diagnostics and therapeutics in lung diseases in childhood and beyond (Figure 1).

## Review

### Chitin

Chitin, a polymer of *N*-acetylglucosamine and the second most abundant polysaccharide in nature following cellulose, is an essential component of fungi, house-dust mites, exoskeletons of crabs, shrimp and insects, parasitic nematodes, and digestive tracts of many insects [1]. Chitin protects these microbes from their environment, and its turnover is regulated by biosynthesis and degradation through endogenous chitinases.

The first immune stimulatory activity of chitin and chitin derivatives in mammals was discovered and extensively explored in the middle to late 1980s, as reviewed recently [2,1]. These early studies clearly indicated that chitin has important immunologic effects *in vitro* and *in vivo*, initially highlighted by Shibata et al. who demonstrated that chitin activates peritoneal/alveolar macrophages and natural killer (NK) cells to express a number of pro-inflammatory cytokines such as interleukin-1β (IL-1β), colony-stimulating factor (CSF), and gamma interferon (IFN-γ) [4]. More recent studies by Reese et al. addressed the *in vivo* immune effects of chitin in mice [5]. They noted that after several hours of chitin exposure, innate immune cells were recruited to the lung and/or the peritoneum. These studies also demonstrated that chitin induced alternative macrophage activation and that macrophage depletion with clodronate liposome treatment prevented the recruitment of eosinophils. Van Dyken et al. further demonstrated that fungal chitin from asthma-associated home environments induced allergic eosinophilic lung inflammation [6]. Collectively, these studies strongly suggest that chitin can contribute to the development of allergic type 2 (Th2) inflammation by activating innate immune cells. Beyond these findings, chitin has also been proposed to serve as an immunoadjuvant. In murine asthma models, Shibata et al. demonstrated that orally administered chitin suppressed the production of Th2 cytokines and IgE [7] and, when used as an adjuvant, chitin caused similar effects as a Th1-promoting adjuvant [8,9]. Accordingly, one can speculate that the immune system senses chitin and that chitin can skew the Th1/Th2 immune response in a bidirectional way.

The studies noted above suggest that the size of the chitin fragment is a crucial determinant of the effector responses that it elicits. This can be seen in comparisons of large chitin polymers that are biologically inert and intermediately sized fragments, which trigger IL-17, IL-23, and TNF-α production [1], while even smaller fragments enhance the production of the anti-inflammatory cytokine IL-10. Therefore, chitin has size-dependent effects on murine immune cell function and may bind to different receptors in a size-dependent manner, similar to findings in plants [10,11]. The observation that large chitin polymers are inert, smaller fragments are pro-inflammatory, and even smaller fragments exert an anti-inflammatory effect allows for an interesting hypothesis regarding the importance of the size-dependent effects of chitin in this response. However, in all of these studies, it is difficult to rule out that chitin preparations contained mixtures of differently long chitin polymers or that ‘large’ chitin preparations were containing contaminating ‘smaller’ chitin fragments. Clearly, a more thorough study using well-defined chitin fragments is warranted. Collectively, it is tempting to speculate that chitin recognition by pattern recognition receptors triggers the induction of chitinases, leading to the generation of small-sized chitin particles that are taken up by the host cell. Recently, fungal chitin was found to dampen inflammation through IL-10 induction mediated by activation of the intracellular receptors NOD2 and TLR9 [12]. Despite these intriguing insights, the precise molecular recognition principles of chitin perception remain incompletely understood. Interestingly, this hypothesis is very similar to the established biology of another polysaccharide, hyaluronin that also serves as an alarm signal after degradation. Thus, the ability of appropriately sized polysaccharides to induce inflammation may be a more general principle of glycobiology [2,1]. When viewed in combination, chitin is a central component of potential pro-allergic microbes (e.g., *Aspergillus fumigatus* and house-dust mite) and has been shown to drive Th2-associated immune responses. Mechanisms that interfere with chitin metabolism are therefore of high relevance for allergic diseases and infections with chitin-bearing pathogens such as fungi.

### Chitinases and chitinase-like proteins

Chitin-degrading enzymes, known as chitinases, are produced by humans and other mammals and are part of the 18-glycosyl-hydrolase family that encompasses both enzymatically active chitinases and chitinase-like proteins, the latter also termed chi-lectins, which lack enzymatic activity. In humans, acidic mammalian chitinase (AMCase), chitotriosidase, oviductin, and human cartilage glycoprotein (HcGP)-39/YKL-40 and YKL-39 have been described, while YM-1, YM-2, AMCase, oviductin, and breast regression protein (BRP-39) have been identified in mice [13,1,14-17]. Humans express two functional chitinases, chitotriosidase (*CHIT1*) and AMCase (*CHIA*) with an acidic pH optimum, both able to degrade chitin polymers. In mammals, chitinases [18,19] and chitinase-like proteins [20] are mainly expressed and secreted by phagocytes (mainly neutrophils and macrophages) and are induced at sites of inflammation, infection, and tissue remodeling, suggesting that these proteins play active roles in anti-infective defense and repair responses. Specifically, both chitinases and chitinase-like proteins have been linked to an alternative activation (M2) phenotype of macrophages [21,1,22], which is found in asthma and other chronic diseases, such as cystic fibrosis (CF), providing a rationale for chitinases and chitinase-like proteins to play a role in these disease conditions. While chitotriosidase is used as disease biomarker for Gaucher disease, a disease characterized by the accumulation of lipid-laden macrophages, acidic mammalian chitinase has been linked to allergic asthma and hypersensitivities [23]. However, the major evidence for an involvement in lung diseases exists for chitinase-like proteins. Therefore, we will focus on their role in pediatric lung diseases in the chapters below.

### The chitinase-like protein YKL-40 and its involvement in lung diseases

YKL-40, or also called HcGP-39 or BRP-39 in mice, lacks measurable enzymatic chitinase activity, due to mutations in its highly conserved putative active sites [17]. In mice, BRP-39 has been associated with cell growth, breast cancer, and tissue remodeling. Recent evidence from BRP-39 knock-out mice indicates that BRP-39 plays a role in T-cell, macrophage and dendritic cell responses as well as cell apoptosis and tissue repair [21,1]. In human cells, HcGP-39/YKL-40 has been shown to regulate apoptosis/proliferation/cell survival, MAPK, and cytokine pathways [24,2].

The first indication that YKL-40 could be linked to human lung diseases came from a multi-center study quantifying YKL-40 serum levels in 253 adult patients with asthma [25]. This study showed that YKL-40 serum levels were mainly increased in adult patients with severe asthma and correlated with disease severity and airway remodeling. These studies in adults were, on the one hand, confirmed for children with severe, therapy-resistant asthma [26], but, on the other hand, challenged by another study [27]. Besides increased levels of YKL-40, elevated *bona fide* chitinase activities were also found in bronchoalveolar-lavage (BAL) fluids from children with asthma [28]. YKL-40 was also found to be increased in BAL fluid after segmental allergen challenge, indicating local production of this chitinase-like protein in response to allergens [29]. Gavala et al. confirmed this finding and further demonstrated that segmental allergen challenge also increased chitinase activities [30]. Clinically, YKL-40 serum levels remained increased in patients in spite of long-term inhaled corticosteroids, which could imply that YKL-40 production is resistant to current asthma treatments and might represent an alternative therapeutic target for severe asthma. YKL-40 is mainly released by activated neutrophils [20], and neutrophilic asthma is well known to be corticosteroid-resistant [25]. Thus, increased YKL-40 levels may be a hallmark of neutrophilic asthma. A follow-up study analyzing single nucleotide polymorphisms (SNPs) in the *YKL-40/CHI3L1* gene showed a genetic association with increased susceptibility to asthma, increased bronchial hyperresponsiveness, and reduced lung function [31]. The role of *YKL-40/CHI3L1* SNPs for asthma was further confirmed in a Taiwanese population [32]. Another study found a different *YKL-40/CHI3L1* variant associated with asthma [33]. Given the enigmatic role of YKL-40, the functional importance of these variants awaits further investigation. Moreover, a Chinese study found that YKL-40 levels were increased in asthmatic patients and correlated with exacerbation, eosinophils, and immunoglobulin E [34], while a study from Poland found increased YKL-40 levels in asthma, but no correlation with disease severity or total IgE levels [35]. Besides a positive correlation between YKL-40 levels and age in subjects with asthma across all age groups, Santos et al. found no difference in circulating YKL-40 levels among asthma severities in children nor a correlation with IgE levels [27]. When viewed in combination, some but not all studies support the notion that YKL-40 is increased in severe and/or neutrophilic asthma, yet the clear relationship with age/disease progression (pediatric vs adult) and atopy remains to be defined in future studies.

Beyond asthma, other pulmonary or lung-associated disease conditions where YKL-40 levels were found to be increased were chronic obstructive pulmonary disease (COPD) [36,37], idiopathic pulmonary fibrosis [38], tuberculous pleural effusions and pneumonia [39], small-cell lung cancer [40], non-small-cell lung cancer [41], bronchiolitis obliterans syndrome [42], hyperoxic acute lung injury [43], sarcoidosis [44], allergic rhinitis [45], and CF lung disease [46]. Despite the heterogeneity of these disease conditions, alternative (M2) macrophage activation is a common feature of the majority of them, suggesting that increased YKL-40 levels reflect M2 macrophage polarization and disease conditions featuring M2 activation [24,22,2,47].

In CF, a chronic neutrophilic inflammatory disease [48,49], YKL-40 BALF levels were found to reflect airway inflammation and infection in early CF lung disease [50] and correlated inversely with lung function in adult CF patients, where YKL-40 levels were also found to be increased systemically [46]. The potential role of YKL-40 for the pathogenesis of CF lung disease is further supported by findings in *Scnn1b*-transgenic mice, a murine model of CF-like lung disease [51,52]. In this model, airway-specific overexpression of the β-subunit of the epithelial Na^+^ channel ENaC mimics airway surface dehydration characteristic for CF airways and produces a CF-like lung disease with early onset of mucus obstruction, chronic airway inflammation, slowed bacterial clearance, and progressive structural lung damage [53]. Similar to patients with CF, levels of the murine homologue of YKL-40, BRP-39, were significantly increased in BALF and showed an inverse correlation with pulmonary function in *Scnn1b*-transgenic mice [46]. Collectively, these studies suggest that YKL-40/BRP-39 may be implicated in the pathogenesis of chronic airway inflammation and airflow obstruction and thus serve as a potential biomarker of disease severity in patients with CF.

Recent studies in this mouse model of CF-like lung disease also provided first mechanistic insights on how chitinases/chitinase-like proteins (CLPs), including BRP-39, may be upregulated in CF lung disease. These studies demonstrated that CF-like airway surface dehydration causing mucociliary dysfunction and mucus obstruction [54,55] provides a robust stimulus for macrophage activation, even when *Scnn1b*-transgenic mice are kept in a germ-free environment [54, 56]. More recently, a series of gene expression studies identified signatures of alternatively activated macrophages (M2), including *Ym1, Ym2,* and *BRP-39,* in whole lungs and isolated macrophages from *Scnn1b*-transgenic mice [57, 58]. These results suggest alternative macrophage activation in mucostatic airways as a mechanism underlying elevated expression of a range of chitinases and CLPs, even in the absence of chitin-containing parasites or allergens, in the airways of patients with CF and potentially other muco-obstructive lung diseases. While the pathogenic role of chitinases/CLPs in (dys)regulation of inflammation in CF airways remains poorly understood, observations in lungs from *Scnn1b*-transgenic mice demonstrate that some proteins of the chitinase/CLP family, such as *Ym1* and *Ym2*, are expressed at such high levels that they precipitate and form sharp crystals often greater than 100 μm in size [54]. These may cause chronic mechanical irritation and injury of airway epithelial cells and phagocytes thus contributing to chronic airway inflammation. Additionally, it is intriguing to speculate that such crystals may activate the NLRP3 inflammasome, a pattern recognition receptor able to trigger inflammation in response to other crystalline or aggregated endogenous substances such as cholesterol or uric acid crystals [59,60,61].

Beyond asthma and CF, circulating YKL-40 has been further associated with decline of lung function in the general population and has been proposed as a biomarker of susceptibility to the long-term effects of cigarette smoking [62]. Studying chitinases in New York City firefighters after World Trade Center exposure revealed that increased serum chitotriosidase reduced the odds of developing pulmonary obstruction after World Trade Center-particulate matter exposure and was associated with recovery of lung function. The underlying mechanisms remain unclear [63]. As chitin is a structural component of fungi, which are well known for their role in environmental asthma, a study investigated whether the exposure to environmental fungi modulates the effect of chitinases in individuals with asthma. The study demonstrated that environmental exposure to fungi modified the effect of *CHIT1* SNPs on severe asthma exacerbations [64].

In order to dissect the cellular sources of YKL-40 in human airways and the mechanisms regulating YKL-40 expression, Park and coworkers identified human airway epithelial cells as a source of YKL-40 and demonstrated that mechanical stress potently induces CHI3L1 expression leading to increased secretion of YKL-40 protein in an EGFR and MEK1/2-dependent pathway, suggesting that mechanical stress contributes to enhanced YKL-40 levels in asthmatic lungs [65]. A further mechanistic study found that YKL-40 increased the proliferation and migration of bronchial smooth muscle (BSM) cells through PAR-2-, AKT-, ERK-, and p38-dependent mechanisms and demonstrated that YKL-40 epithelial expression was positively correlated with BSM mass in asthma [66]. Other studies showed that YKL-40 induced IL-8/CXCL8 expression from bronchial epithelium via MAPK (JNK and ERK) and NF-κB pathways [67]. Taken together, these results suggest that YKL-40-mediated IL-8 production could be related to BSM remodeling [68]. Further studies showed that the allergen ovalbumin increased YKL-40 expression in tracheal epithelial cells [69] and demonstrated that YKL-40 increased mucin5AC production in human bronchial epithelial cells [70]. Collectively, these studies extend the view that YKL-40 is mainly a marker of neutrophilic inflammation by demonstrating modulatory effects of this chitinase-like protein on airway epithelial cells. Despite these intriguing insights into the biological effects of chitinase-like proteins, their precise functional role in biological processes and disease conditions in humans still remains largely unclear.

## Conclusions

Chitin, chitinases, and chitinase-like proteins remain enigmatic terms for human diseases. However, after the second look into the pathophysiology of allergic and chronic lung diseases, these ancient‚ insect glycoprotein-associated pathways attract high relevance as potential biomarkers and therapeutic targets. Particularly, fungal chitin-linked asthma is increasing, but treatment options and successful clinical trials are scarce, necessitating further therapeutic developments. Before those approaches can be applied clinically, several key questions remain to be answered:

- How is chitin recognized by the human immune system? Is it a novel pattern recognition receptor ligand? And if so, can this interaction be targeted and exploited therapeutically?
- What is the primary role of enzymatically active chitinase found in the human body? To defend against chitin-bearing pathogens or to skew the immune system?
- Is a dysregulation of chitin sensing or YKL-40 induction pathways associated with altered susceptibility for diseases like fungal asthma or house-dust mite allergy?
- Is YKL-40 a potential biomarker of severe asthma, a marker of neutrophilic innate immune activation, or a marker reflecting tissue remodeling (or all of these)?
- Does YKL-40 play a causative role in these disorders? Can YKL-40 be neutralized pharmacologically? And if so, which diseases would benefit?
- Considering YKL-40 as a therapeutic target in human diseases, what is the physiologic function of this protein?

These issues remain to be solved and pave the way for an exciting novel field in pediatric immunology, bridging a gap between insects, fungi, immune cells, and the lung.
